# Removal of the Uterine Appendages for the Arrest of Uterine Hemorrhage

**Published:** 1882-02

**Authors:** 


### REMOVAL OF THE UTERINE APPENDAGES FOR THE ARREST OF UTERINE HEMORRHAGE.

---

In the *American Journal of Medical Sciences*, January, 1882, Mr. Lawson Tait, the distinguished English ovariotomist, reports thirty-one cases in which the uterine appendages (ovaries and Fallopian tubes) were removed, for otherwise incurable uterine hemorrhage. The causes of the hemorrhage are as follows:

| | |
|---|---|
| Myoma of the Uterus, - - - - - | 20. |
| Cystoma of Ovaries, - - - - - - | 6. |
| Ovaritis, - - - - - - - - - | 3. |
| Cysts of Fallopian Tubes, - - - - | 1. |
| Cirrhosis of Ovary, - - - - - - | 1. |

There were four deaths, all in cases in which the operation was performed for myoma. In twenty one cases complete arrest of the hemorrhage is reported, and three cases are announced as being greatly improved. Of the last twenty-five cases two died. In this last series the spray with the details of the Listerian ceremonial were used in thirteen cases with one death; twelve cases without the spray, one death.

Mr. Tait, at the end of his article, draws the following conclusions:

1st. That as far as its primary results are concerned, removal of the uterine appendages for the arrest of intractable uterine hemorrhage, is an operation which is quite as easily justified as any of the major operations of surgery.

2d. That as far as its secondary results are yet known, it is an operation which yields abundant encouragement for its further trial.

As conclusions which are indicated but not wholly proved, I think I may formulate a statement that removal of the ovaries alone is not sufficient to arrest menstruation, but that removal of both tubes and ovaries does at once arrest it. So far as some of these cases have gone, the arrest would seem to be permanent. This conclusion is quite in harmony with what is known of removal of both ovaries for large cystomata, for in such cases the tubes are almost uniformly included in the clamp or ligature, and menstruation is arrested.

Three at least of the cases, and probably two others, show that the arrest of menstruation by this means leads, or may lead, to the atrophy of the tumors.

Finally there is some connection here pointed out I believe for the first time, and worthy of very clear study, between uterine myoma and its accompanying hemorrhages, and cystic disease of the ovaries. In two of the cases the cystic disease seemed to be the cause of the hemorrhage, without any myoma intervening

Another conclusion is, I think to be justified, that the whole subject is worthy of careful study, and should not be made the subject of premature and hostile conclusions.

